# Dental conditions associated with preventable hospital admissions in Australia: a systematic literature review

**DOI:** 10.1186/s12913-018-3733-2

**Published:** 2018-12-03

**Authors:** Abhinav Acharya, Shahrukh Khan, Ha Hoang, Silvana Bettiol, Lynette Goldberg, Leonard Crocombe

**Affiliations:** 1 Centre for Rural Health, College of Health and Medicine, University of Tasmania, Hobart, Tasmania Australia; 2 Centre for Rural Health, College of Health and Medicine, University of Tasmania, Launceston, Tasmania Australia; 3 School of Medicine, College of Health and Medicine, University of Tasmania, Hobart, Tasmania Australia; 4 Wicking Dementia Research and Education Centre, College of Health and Medicine, University of Tasmania, Hobart, Tasmania Australia

**Keywords:** Hospitalisation, Oral health, Dental, Australia, Public health

## Abstract

**Background:**

Over the past two decades, there has been a decrease in dental diseases in Australia; however, the number of preventable dental hospital admissions has not diminished. This review reports on the factors associated with preventable dental hospital admissions in Australia.

**Methods:**

A search of five databases was conducted using Medical subject headings/Emtree terms and Index terms. All original studies, published between January1965 and March 2018 in English, based on the Australian population, and examining the prevalence of oral conditions as a cause for emergency department presentations and hospital admissions were included. The mixed method appraisal tool was used to evaluate the included studies.

**Results:**

Eleven cross-sectional studies met inclusion and exclusion criteria. All the studies, except one from Tasmania, were from Western Australia. The most common reasons for preventable dental hospital admissions were dental caries, followed by embedded or impacted teeth. Malignant neoplasms were reported as main causes of preventable dental hospital admissions in the older population.

**Conclusions:**

Most studies on preventable dental hospital admissions were from one Australian state (Western Australia). Further research is required to determine the national prevalence and incidence of preventable dental hospital admissions. A periodic audit of preventable dental hospital admission data is needed for delivery of a fair and effective dental services.

## Background

Potentially Preventable Hospital Admissions (PHA) are defined as conditions that could be avoided for hospitalisation if timely management and primary care had been provided [1]. PHA is a health system performance indicator of the accessibility to and effectiveness of primary health care, providing information on socioeconomic and racial disparities in access to primary health care, health inequalities and literacy, and the prevalence of health conditions in the community [1, 2]. Dental conditions, which are categorised as acute medical conditions, are one of the components of PHA according to the National Healthcare Agreement 2016 between the State and Commonwealth Governments of Australia [3].

There has been marked reductions in dental conditions in adults (tooth loss and dental decay) observed in two national surveys of adult oral health completed in 1987–88 and 2004–06 [4]. The reduction of dental caries in adults could be a result of reduction in tobacco consumption, adoption of better oral hygiene practices and public health interventions (water fluoridation and interventional procedures) [5], and provision of adult dental benefit schemes [6]. Time trend report suggests that dental caries experience among children 5–6 years and > 12 years declined significantly from 1970s until the late 1980s. However, the latest National Child Oral Health Survey (NCOHS) 2012–14 showed that the dental caries experience has been in fluctuation since 1987–88 survey, suggesting no serious decline in the dental caries experience in between the two time points [7].

In contrast to the above findings, the Preventable Dental Hospital Admissions (PDHA) have not diminished. Dental conditions contribute to 9 % of total PHA in Australia [8]. There were 57,955 cases of dental conditions leading to PHA in 2007–2008, which had increased to 63,456 in 2013–2014 [9].

Hence, to develop a sound understanding of dental causes that contributes towards PDHA, it is important to map the factors that may lead to preventable PDHA in the Australian population. This study aimed to synthesise the literature on the factors that lead to PDHA in Australia. This review will assist policy makers and oral health practitioners to understand the causes of PDHA and find methods to prevent PDHA, thereby reducing costs associated with PDHA.

## Methodology

### Review question

The Population Intervention Comparator Outcome (PICO) criteria and the Preferred Reporting Items for Systematic Reviews and Meta-Analyses (PRISMA) guidelines were used to devise and construct the review question. The review question was “What are the main causes of PDHA in Australia?”

### Scope of review and search terms

Published results of studies examining the prevalence of oral / dental conditions as a cause for emergency department presentations and hospital admissions were reviewed. Articles were retrieved using searches performed in PubMed/MEDLINE, EMBASE, Web of Science, CINAHL and Google Scholar. The search terms used in the review were generated using the National Library of Medicine Medical Subject Headings (MeSH) terms, Emtree terms and free text terms. Table 1 shows the search terms used in the systematic review.

**Table 1:** Search Terms

Mesh Terms for oral conditions	Free text terms	Mesh terms for Hospitalization
Periodontal disease	Dental condition	Hospitalization
Periodontitis	Oral Condition	Institutionalization
Chronic Periodontitis	Adult Periodontitis	Preventable hospitalization
Temporomandibular Joint Disorders	Dental abscess	Emergency service
Dental caries	Oral abscess	Emergency Medicine
Jaw fracture	TMJ Diseases	Emergency Medical Services
Mandibular fracture	TMJ Disorders	Patient admission
Maxillary fracture	Temporomandibular Disorder	Patient readmission
Maxillofacial injuries	Oral cancer	Emergencies
Facial injuries	Oral squamous cell carcinoma	First Aid
Mouth Diseases	Tooth decay	Crisis Intervention
Halitosis	Dental decay	ER
Pulpitis	Tooth pain	Hosp
Tooth Avulsion	Dental pain	ED
Facial Nerve Diseases	Tooth fracture	Emergency Department
Trigeminal Nerve Diseases	Dental fracture	Length of stay
Facial Neuralgia	Maxillofacial injury	Emergency
Lingual Nerve Injuries	Oral diseases	
Mouth Neoplasms	Oral Manifestations	
Oral Fistula	Oral health	
Oral Ulcer	Endodontic Inflammation	
Oral Submucous Fibrosis	Pulpitides	
Ranula	Endodontic infection	
Salivary gland Diseases	Tooth Avulsions	
Xerostomia	Facial Nerve Injury	
Stomatitis	Facial Nerve Trauma	
Tongue diseases	Facial Neuropathy, Traumatic	
Oral Tuberculosis	Cranial Nerve VII Injuries	
Glossitis	Facial Nerve Avulsion	
Hairy Tongue	Tooth Luxation	
Tongue Neoplasms	Avulsed Tooth	
Sialadenitis	Tooth Luxation	
Aphthous Ulcers	Tooth Dislocation	
Herpetic Stomatitis	Dental implant failure	
Denture Stomatitis		
Peri-implantitis		
Root caries		
Dental Fluorosis		

### Inclusion and exclusion criteria

All original studies addressing the research question, published between January 1965 and March 2018 in English and based on the Australian population, including both adults and children, were included in the review. Studies were excluded if they were conducted outside Australia or were reviews, conference papers, short communications, letters or textbooks.

### Quality appraisal

The Mixed Method Appraisal Tool (MMAT) was used for quality appraisal of the selected articles [10]. All of the articles included in the study were cross sectional studies, which fall under quantitative non-randomised study. Two independent reviewers (A.A. and H.H.) assessed the six criteria designed to evaluate the quality of methodology and overall quality score was calculated. All of the articles fulfilled methodological quality criteria as per MMAT. Table 2 shows the criteria and the summary of MMAT.

**Table 2:** Quality appraisal of studies using Mixed Method Appraisal Tool

Author and year	Are there clear quantitative research questions (objectives)?	Do the collected data address the research question (objective)?	Are participants recruited in a way that minimises selection bias?	Are measurements appropriate regarding the exposure/intervention and outcomes?	In the groups being compared, are the participants comparable, or do researchers take into account the difference between the groups?	Are there complete outcome data (80% or above), or an acceptable follow-up rate for cohort studies (depending on the duration of follow up)?	Overall quality score
Tennant et.al., 2000 [16]	yes	yes	yes	yes	yes	yes	1
Smith et.al, 2006 [17]	yes	yes	yes	yes	yes	yes	1
Kruger et.al, 2006 [15]	yes	yes	yes	yes	yes	yes	1
Slack-Smith et.al, 2008 [12]	yes	yes	yes	yes	yes	yes	1
Slack-Smith et.al, 2011 [13]	yes	yes	yes	yes	yes	yes	1
Slack-Smith et.al, 2012 [11]	yes	yes	yes	yes	yes	Yes	1
Verma et.al, 2014 [10]	yes	yes	yes	yes	yes	Yes	1
Alsharif et.al,2014 [14]	yes	yes	yes	yes	yes	Yes	1
Kruger et.al, 2015 [20]	yes	yes	yes	yes	yes	Yes	1
Kruger et.al, 2016 [19]	yes	yes	yes	yes	yes	Yes	1
Kruger et.al, 2016 [18]	yes	yes	yes	yes	yes	Yes	1

## Results

### Selection of studies

**Fig. 1:**
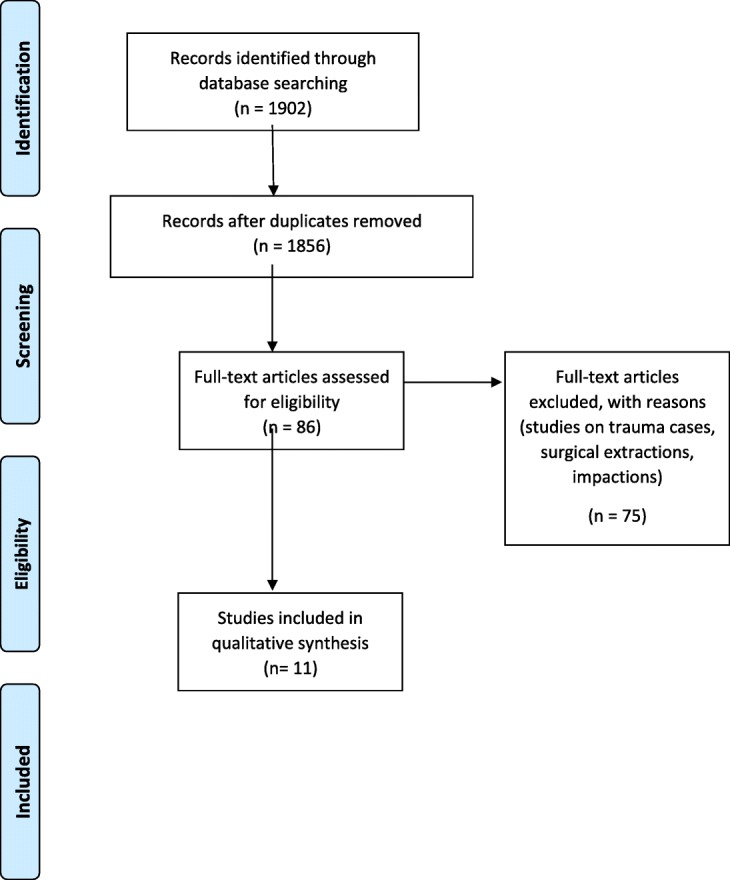
PRISMA flow diagram of literature search and paper selection process

The literature search and removal of duplicates identified 1856 papers as eligible for inclusion in the review. These studies underwent title and abstract screening independently by two reviewers (A.A and S.K), resulting in selection of 86 studies for full text review. After the full text review, 11 studies met the inclusion and exclusion criteria and were included in the table of synthesis (Table 3). The PRISMA diagram shows the flowchart of selection of studies in the systematic review (Fig. 1).

**Table 3:** Characteristics of studies and the summary of results

Author, year, state	Number of hospitalised episodes/age	Classification for diagnosis of disease	Study design	Covariates	Summary of results	
					Reason for hospitalisation	Demographic factors
Tennant et.al., 2000 [16] WA	*N* = 3754 Age (0-17 Years) 1. Infant (0-1y) 2. Preschool (1-4 yrs) 3. Primary school (5-12 yrs) 4. High school (13-17 yrs)	ICD 9 [mentioned as ICD (5200–5299)]	Cross sectional Retrospective	Aboriginality, Residency (rural and metropolitan), Age, Gender	Dental caries was the primary reason for hospitalisation in preschool and primary school children. Abnormal tooth eruption was the primary reason for hospitalisation in high school children.	Out of total hospitalised cases, • 0.6% were infants, • 22.3% pre-schoolers, • 25.7% primary school children and • 51.5% high school children. Males (44%) and non-Aboriginal decent (98%) were main groups for dental related hospital admissions Non-aboriginal high school children and infants had higher hospitalisation due to oral condition as compared to Aboriginals. Rural child had 1.3 times higher risk of hospitalisation due to dental condition as compared to metropolitan.
Smith et.al, 2006 [17] WA	*N* = 53,646 Age Adult population (18–85+ years)	ICD- 10 AM	Cross sectional Retrospective	Age, Indigenous status, Residency (rural and metropolitan), IRSD, ARIA	The prevalence of hospital admissions due to oral conditions were: • Embedded and impacted teeth (38.8%) • Dental caries (8%). After excluding embedded and impacted teeth the main reason for hospitalisations were • Dental caries • Maxillary sinusitis • Malignant neoplasms (oral related) • Disorders of teeth and supporting structures.	Female (52.2%) were more hospitalised than male. Most common reason for hospitalisation with age • Individuals less than 35 years -gingivitis and periodontal diagnosis, 17.5% • Individuals 35 years and older- malignant neoplasms, 14.5% Indigenous Australians were admitted 1.5 times more in hospital for oral health conditions (*p* ≤ 0.05). Least disadvantaged people (*p* ≤ 0.05) and highly accessible people (*p* ≤ 0.05) were more likely to be hospitalised due to oral health related conditions.
Kruger et.al, 2006 [15] WA	*N* = 26,497 Age (0-17 Years) 1. 0-1y 2. 1-4 yrs. 3. 5-12 yrs. 4. 13-17 yrs	ICD- 10 AM	Cross sectional Retrospective	Age, Aboriginality, Residency, Gender,	The reasons for hospitalisation were • Embedded and Impacted teeth – 33.2% • Dental Caries- 28.3% • Pulp and peri-apical tissues – 7.1% • Dental facial anomalies − 6.1% • Birth trauma and congenital deformities – 4.1%	Out of total oral condition related hospital admission cases, 50.2% were male and 3.5% were aboriginal descent. The number of hospitalisation was more in rural aboriginal children as compared to urban non aboriginals.
Slack-Smith et.al, 2008 [12] WA	*N* = 11,523 Age (0–5 years)	ICD – 9	Cross sectional Retrospective	Age, Sex, Birth weight, year of birth, SEIFA, Health insurance, Health region, Rurality, Maternal age group, Mother’s Indigenous status, Intellectual disability, Birth defect	The reasons for hospitalisation were • Disease of hard tissues of teeth 76.3%, • Disorders of tooth development and eruption 3.7% • Diseases of pulp and periapical tissues 10% • Gingival and periodontal diseases 1% • Dentofacial anomalies including malocclusion 0.2% • Other diseases and conditions of the teeth and supporting structures 0.7% • Diseases of the jaws 0.4% • Diseases of the salivary glands 1.2% • Diseases of the oral soft tissues excluding lesions specific • for gingiva and tongue 5.4% • Diseases and other conditions of the tongue 0.3% • Fitting devices and special investigations 1%	Children (0-5 years) accounted 3% of total dental hospital admission. Logistic regressions showed significantly higher hospitalisation among children (*p* < 0.05) with ˗ birth defect (OR 1.85, CI 1.68–2.05), ˗ Male gender, (OR 1.16, CI 1.08–1.25), ˗ Indigenous mother (OR 1.17, CI 1.02–1.34), ˗ No water fluoridation (OR 2.16, CI 1.94–2.40) ˗ Intellectual disability, ˗ privately funded health insurance
Slack-Smith *et.al,* 2011 [13] WA	*N* = 738 Age (0–5 years)	ICD-9, (ICD-10 AM was converted to ICD-9 for individuals admitted after 1-07-1999).	Cross sectional Retrospective	Indigenous status, Age, ARIA, Length of stay	Main causes of dental admission were: • Disorders of tooth development and eruption 4.16% • Diseases of hard tissues of teeth 37.67% • Diseases of pulp and periapical tissues 10.98% • Gingival and periodontal diseases 5.28% • Other diseases and conditions of the teeth and supporting structures 0.14% • Diseases of the jaws 0.54% • Diseases of the salivary glands 2.57% • Diseases of the oral soft tissues excluding lesions specific • for gingiva and tongue 35.64% • Diseases and other conditions of the tongue 1.22% • Fitting devices and special investigations 0.81% • Dental examination 0.54%	3.2% of indigenous children had dental related hospital admission as compared to 2.7% non-indigenous children. Indigenous children had more dental related hospital admission at age less than 2 years as compared to non-indigenous children. (40% versus 10%, *P* < 0.0001). 6.3% of total dental related hospital admission were indigenous children. Out of total indigenous children dental admission, 8.7% had birth defect and 5.5% had intellectual disability. Length of stay (7 days or more) in hospital was recorded higher in indigenous versus non-indigenous children (11.2% versus 0.5%, *P* < 0.001). Remoteness (OR 2.07 versus 1.05), public funded assistance (89% versus 44%), rural residence (OR 9.61 versus 1.48) were significantly associated factors with dental related hospital admissions in Indigenous children versus non-indigenous children.
Slack-Smith *et.al,* 2012 [11] WA	*N* = 1513 0–2 years	ICD-9 (ICD-10 AM was converted to ICD-9 for individuals admitted after 1-07-1999).	Cross sectional Retrospective	Age, Intellectual disability, Length of stay, Child year of birth, Sex, Mother’s Indigenous status, SEIFA, Health insurance, Mothers age group, Rurality, Health region, Birth defect.	Reasons for dental related hospital admissions; • Disorders of tooth development and eruption 10.7% • Diseases of hard tissues of teeth 38.9% • Diseases of pulp and periapical tissues 5.9% • Gingival and periodontal diseases 4.4% • Dento-facial anomalies including malocclusion 0.3% • Other diseases and conditions of the teeth and supporting structures 1.3% • Diseases of the jaws 1.8% • Diseases of the salivary glands 4.6% • Diseases of the oral soft tissues excluding lesions specific • for gingiva and tongue 29.4% • Diseases and other conditions of the tongue 1.5% • Fitting and adjustments of other devices-orthodontic devices 0.4% • Special investigations and examination 1.0%	Male gender (OR 1.14), low birth weight (OR 1.17), birth defects (OR 1.74), intellectual disability (OR 2.10), children of indigenous mother (OR 4.45), having public health insurance (OR 1.29), and rurality/remoteness (OR 2.29) had significantly higher odds of dental related hospital admissions. Least disadvantaged has significantly lower risk of dental related hospital admission than most disadvantaged (OR 0.58).
Verma *et.al,* 2014 [10] Tasmania	*N* = 454 0–86 years (mean age 32)	N/A	Cross Sectional Retrospective	Age, Gender, timing of presentation	Causes for attending ED due to dental related cause: • Dental abscess 37.2% • Toothache 31.5% • Dental caries 8.8% • Tooth fracture 7.3% • Tooth avulsion or loss 6.8% • Gingivo-stomatitis 4.6% • Aphthous ulcer 3.1% • Temporomandibular joint Disorder 0.7%	Male (60.2%) had more dental presentation to ED than female (39.8%). Individuals with ages 26–30 years had highest dental presentations in ER (17%). Average age of patients with dental abscess (the most common presentation) was 36.59 years. 68% of presentation were out of business hours.
Alsharif*et.al*,2014 [14] WA	*N* = 43,937 0–14 years	ICD 10 AM	Cross sectional Retrospective	Hospital area and type, Age, gender, Indigenous status, SEIFA, ARIA, Insurance status, Length of stay	Major categories of dental related hospital admissions: • Dental caries 50% • Embedded and Impacted teeth 14% • Pulp and periapical tissue conditions 11% • Developmental and birth defects 5% • Dental Fractures 5% • Dentofacial anomalies 4%	5% of total dental related hospital admission for this age group were indigenous children. 73% admissions were in less than 9 years old age group. Rate of admission in children age 5–9 years increased significantly as compared to age group 0-4 years in 2009. Non indigenous children were more likely to be admitted for all dental causes except pulp and periapical conditions, and dental fractures. Males, least disadvantaged, high accessibility and uninsured children had significantly more dental admissions (*p* < .0.001). The dental related hospital admissions were more in public (*p* < 0.001) and metropolitan hospitals.
Kruger *et.al,* 2015 [20] WA	*N* = 65,005 0–75+ years	ICD-10 AM	Cross Sectional Retrospective	Age, gender, ethnicity, SEIFA, Indigenous status, ARIA, AR-DRG, Income, Housing, Education, Employment, Family structure, Disability, Transport.	The rate of hospitalisation due to preventable dental cause has been increasing significantly over the years. • Dental caries 53% • Other disease of hard tissue of teeth 1.2% • Pulp and periapical tissue 14.3% • Gingivitis and periodontal disease 5.1% • Other gingival and edentulous alveolar ridge 0.6% • Other disorders teeth and supporting structures 18.1% • Cysts of oral region 1.9% • Stomatitis and related lesions 2.2% • Other diseases lip and oral mucosa 3.6%	3.2 per 1000 people were admitted to hospital due to oral condition. Females, aboriginals, most disadvantaged, highly accessible people had more hospital admission due to oral conditions.
Kruger & Tennant 2016 [19] WA	N = 11,608 65 years and older	ICD-10 AM	Cross sectional Retrospective	Age, gender, ethnicity, SEIFA, Indigenous status, ARIA, AR-DRG, Income, Housing, Education, Employment, Family structure, Disability, Transport.	Causes of dental related hospital admission: • Malignant neoplasms 16.6% • Dental caries 15.4% • Other disorders of teeth and supporting structures 14.3% • Other diseases of the jaws 11.3% • Fractures of the teeth, nasal bone, palate, lower facial bones 5.4% • Benign neoplasms 5.4% • Embedded and impacted teeth 5.1% • Diseases of salivary glands 4.5% • Diseases of pulp and periapical tissues 3.8% • Other disease of lip and oral mucosa 3.1% • Stomatitis and related lesions 2.3% • Diseases of the tongue 1.9% • Others 10.8%	Most patients for dental related hospital admissions were from least disadvantaged (27.9%, OR 60.04) and accessible areas (16.4%, OR 10.58). Age 75–79 years (OR 4.7 CI 4.57–4.98), Males (OR 5.82, CI 5.67–6.21) and Aboriginals (OR 9.38, CI 7.82–10.94) had significantly higher rate of hospitalisation (*p* < 0.05).
Kruger *et.al,* 2016 [18] WA	*N* = 131,509 18 years and older	ICD-10 AM	Cross sectional Retrospective	Age, gender, ethnicity, SEIFA, Indigenous status, ARIA, AR-DRG, Income, Housing, Education, Employment, Family structure, Disability, Transport.	Causes of dental related hospital admissions: • Embedded and impacted teeth 48.9% • Dental caries 9.0% • Other disorders of teeth and supporting structures 8.5% • Jaw fractures (maxillary and mandibular) 4.9% • Fractures of teeth, nasal bone, palate, lower facial bones 4.3% • Malignant neoplasms 3.9% • Diseases of pulp and periapical tissues 3.4% • Other diseases of jaws 3.2% • Dentofacial anomalies, including malocclusion 2.6% • Gingivitis and periodontal diseases 2.3% • Others 8.9%	48% were male and 2.8% were aboriginal descent. Rates of admissions were significantly higher in aboriginal as compared to non-aboriginal during 10 year period (*p* < 0.05) The rate of admission was highest for age group 18-29 years for both aboriginal and non-aboriginal.

*ICPC* International Classification of Primary Care, *AR-DRD* Australian Refined Diagnosis Related Groups, *ICD* International Classification of Diseases

*IRSD* Index of relative socio economic disadvantage, *ARIA* Accessibility/Remoteness Index of Australia, *DRG* Diagnostic Related Group, *SEIFA Socio-Economic Indexes for Areas, WA Western Australia*

### Study characteristics

All the studies were from Western Australia (WA) except one study, which was based in Tasmania [11]. Of all the studies, there was one study on age ranges 0–2 years [12], two among 0-5 years [13, 14], one in 0–14 years [15], two in 0–17 years [16, 17], two in 18 years and above [18, 19], one in 65 years and above [20] and two from all age groups [11, 21]. All studies were cross-sectional analysis.

Of the WA studies, six used data from Western Australian Hospital Morbidity Data System (WAHMDS) [15, 16, 18–21]. Two used data collectively from multiple sources: WAHMDS, WA Maternal and Child Health Database for children born in WA, and the WA Intellectual Disability Database and Midwives Notification System Registrar General Database [13, 14]. One study used data from WAHMDS, WA Maternal and Child Health Database for children, and WA Intellectual Disability Database [12]. One used data from the Health Department of WA database [17]. The study in Tasmania has reported on patients presenting with dental conditions to the Emergency Department of the Royal Hobart Hospital [11].

In all studies except one [11], the definition used to identify the PDHA was based on the International Classification of Diseases (ICD), however the ICD versions varied across the studies based on the year published and the update of the classification. Four used ICD 9 [12–14, 17], and six used ICD 10 [15, 16, 18–21]. The Tasmanian study did not report the classification system used to identify the dental causes stating that patients were allocated a code referring to their main complaint [11].

### Dental reasons for PHA

Five out of the eleven studies included in the review identified diseases of the hard tissues of teeth particularly dental caries as the most common cause leading to PDHA in the Australian population [12–15, 21]. The second most common cause of PDHA, reported in three studies, was embedded and impacted teeth [16, 18, 19]. One study listed both dental caries and embedded and impacted teeth as the main cause of PDHA [17]. Malignant neoplasms were reported as main causes of PDHA in one study which investigated PDHAs in the older population [20]. In the Tasmanian study, dental abscesses were the main causes of PDHA [11].

### Demographic factors

Additional factors that accounted for PDHA were indigenous population and aboriginal status [14, 18, 20], children living in areas without water fluoridation [13], people with intellectual disability [12–14], and children of indigenous mothers [13], individuals on public funded assistance [14], remoteness and rural residence [12, 16] (Table 3).

## Discussion

A total of 11 studies were included in this systematic review designed to map the PDHA in the Australian population. Other than the study in Tasmania [11], all the studies were from one state in Australia, WA. This indicates that further research is needed to ascertain the PDHAs across all states and territories of Australia to understand the causes of PDHA, and to find methods to prevent PDHA thereby reducing costs associated with PDHA.

The most common reasons for PDHA across age ranges were dental caries followed by embedded or impacted teeth. Prevention of embedded or impacted teeth is difficult and treatment focuses on their identification and removal. Unfortunately, incidence of impacted teeth may increase as fewer teeth are lost from dental diseases. Preventively however, the high incidence of PDHA for dental caries is a concern. Proper oral hygiene, low sugar consumption, fluoride applications and routine dental visiting are strategies to prevent dental caries [22]. In addition, other preventative strategies recommended by the World Health Organisation [23] include water fluoridation, using fluoride toothpaste and salt fluoridation. There is a need to better prevent and reduce the incidence of dental caries as this will ultimately help to decrease PDHA.

In Australia the prevalence of dental caries is 25.5% in adults and 45–60% in children [24]. Low education, low income, rural location, having no insurance, risk behaviours such as smoking and alcohol, poor dental visiting behaviour and oral hygiene practices, and no fluoride in water are important risk factors associated with dental caries and, if not addressed, subsequent hospitalisation [25]. Similar findings have been reported in New Zealand. In a 20-year review of potentially preventable admissions to public hospitals for dental care, data suggest that dental caries is the most common cause for PDHA in New Zealand [26].

**Fig. 2:**
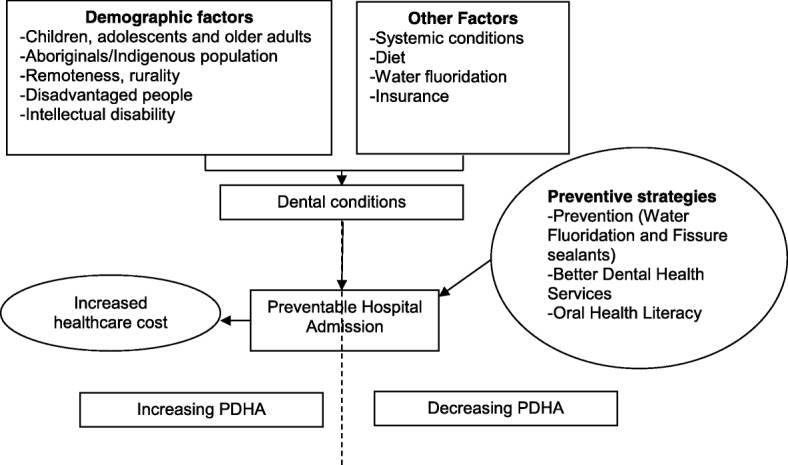
Schematic diagram of preventable dental hospital admission: risk factors and preventive strategies

A preventable hospital admission is a measure of health care accessibility and effectiveness [3]. Hospital admissions due to dental conditions indicate the burden of dental conditions and their associated costs on the health care system [19]. Strategies that could reduce PDHA include (i) preventive dental practices such as water fluoridation and interventional procedures (fissure sealants and fluoride varnish) in public and private healthcare settings [27]; (ii) improved and better access to dental care (child and adult dental benefit schemes) across urban, rural and remote communities [6]; and (iii) oral health literacy and education programs across the population with the use of social media, education and primary health care network platforms. Figure 2 illustrates the schematic representation of risk factors and preventive strategies to prevent PDHAs.

In the Tasmanian study, dental abscesses were the main causes of PDHA [11]. This makes sense because this study investigated dental emergencies presenting to a Royal Hobart hospital emergency department, where people could be expected to present with pain or infections. Important to note was that 68% of all the presentations to the emergency department were after hours, when dentists are unavailable. Hence it is important to consider an after hour dental service for any emergency dental presentation.

Malignant neoplasms were reported as a leading causes of PDHA in the older population in one of the studies [20]. This also does not come as a surprise because the research in this study was limited to people over the age of 65, the age group with the highest incidence of neoplasms [28, 29].

PDHA were higher in indigenous population and aboriginal status [14, 18, 20]. Low education, lower annual income, rural and remote location, having no dental insurance, poor access to care, lack of transportation and unemployment are the associated factors with indigenous people [24]. As oral conditions are associated with systemic health conditions such as obesity and cardiovascular disease, unawareness of maintaining proper oral health by socially disadvantaged people might have negative impacts on their general health [30, 31]. This study will help policy makers to prioritise the preventive approach to address the oral health of socially disadvantaged groups and consequently minimise the PDHA among this group.

The main limitation of this systematic review was the small number of published studies investigating PDHAs in Australia. Of the included studies, almost all were from one state of Australia indicating that further research is needed in PDHA across Australia. Databases with this potential information are available in each state. Investigators need to be encouraged to access, analyse and publish this information as an important preventative and public health issue. There was a high level of heterogeneity observed among the 11 included studies. This limited the authors from conducting a meta-analysis. The studies had a wide variation among two main factors: (i) age; and (ii) the ICD definitions used to define dental conditions that could have been employed in a meta-analysis to predict the effect size of the PDHA across the studies.

PDHA are expensive and they may be reduced by the impartial distribution of public dental clinics [32]. The extension of dental services into areas currently not receiving dental care will not only reduce cost associated with PDHA, but will improve access to oral health care. A periodic audit of PDHA is needed to mobilise government policy-makers and dental service providers for delivery of a fair and effective dental service for all Australians throughout their life course.

## Conclusion

Dental caries is the most common cause of PDHA based on the included studies. As most of studies included are from WA, there is the need of Australia wide research to represent real scenario of PDHA.

## Abbreviations

ARDRD: Australian Refined Diagnosis Related Groups
ARIA: Accessibility/Remoteness Index of Australia
DRG: Diagnostic Related Group
ICD: International Classification of Diseases
ICPC: International Classification of Primary Care
IRSD: Index of relative socio economic disadvantage
MMAT: Mixed Method Appraisal Tool
NCOHS: National Child Oral Health Survey
PDHA: Preventable Dental Hospital Admissions
PHA: Preventable Hospital Admissions
PICO: Population Intervention Comparator Outcome
PRISMA: Preferred Reporting Items for Systematic Reviews and Meta-Analyses
SEIFA: Socio-Economic Indexes for Areas
WA: Western Australia
WAHMDS: Western Australian Hospital Morbidity Data System
